# Supplementary material for: HIV-1 Gag Binds the Multi-Aminoacyl-tRNA Synthetase Complex via the EPRS Subunit
Source: Viruses. 2023 Feb 8;15(2):474. doi: 10.3390/v15020474 (PMC9967848; doi:10.3390/v15020474)
Supplement: Supplementary file 1 [file viruses-15-00474-s001.zip › viruses-2135152-supplementary.pdf]

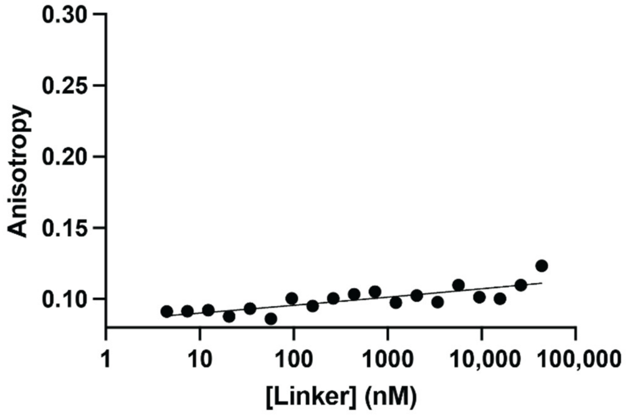

Figure S1. Fluorescence anisotropy binding assay of EPRS linker titrated into fluorescently labeled HIV-1 MA. EPRS linker was titrated into 10 nM Alexa Fluor 488-labeled HIV-1 MA. No significant change in anisotropy was observed in the 0–20 µM of EPRS linker protein titrated.

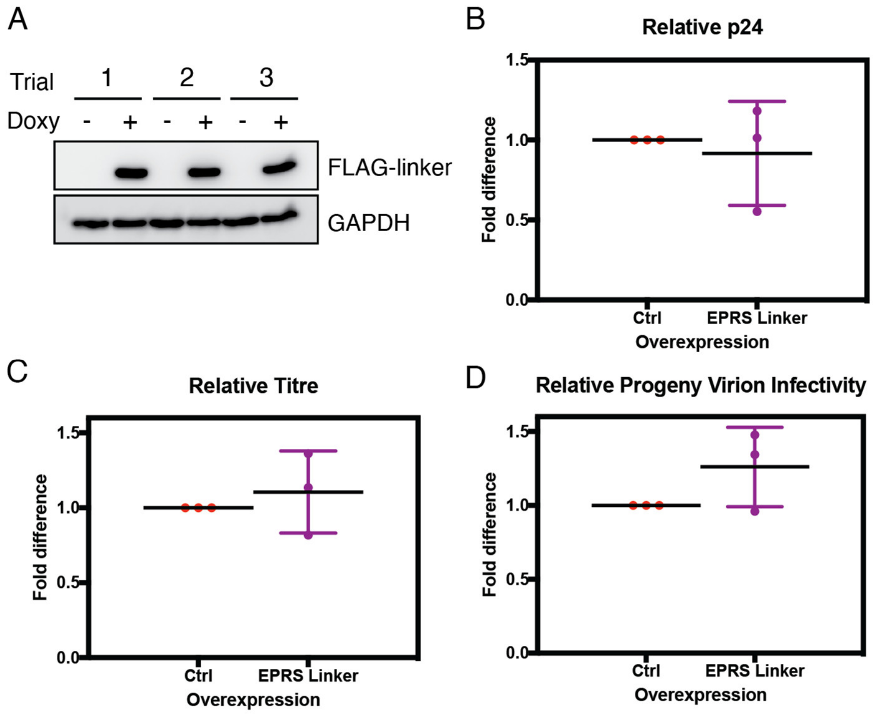

Figure S2. Progeny virion production, titer and infectivity in HEK293T stable cells inducibly expressing EPRS linker-FLAG. (**A**) Expression of FLAG-linker in the presence of doxycycline. (**B**) p24 level in supernatants, measured by HIV-1 p24 ELISA. (**C**) Viral titer measured by limited dilution in GHOST indicator cells. (**D**) Normalized progeny virion infectivity. Three independent experiments were performed in each case. The values of p24 level, titer and relative infectivity for EPRS linker-expressing samples were normalized to their corresponding control samples and presented as fold changes.
